# Probing the effect(s) of the microwaves’ electromagnetic fields in enzymatic reactions

**DOI:** 10.1038/s41598-019-45152-9

**Published:** 2019-06-20

**Authors:** Satoshi Horikoshi, Kota Nakamura, Mikio Yashiro, Kanae Kadomatsu, Nick Serpone

**Affiliations:** 1 Department of Materials and Life Sciences, Faculty of Science and Technology, Sophia University, 7-1 Kioicho, Chiyodaku, Tokyo, 102-8554 Japan; 2 PhotoGreen Laboratory, Dipartimento di Chimica, Universita di Pavia, Via Taramelli 12, Pavia, 27100 Italy

**Keywords:** Biophysical chemistry, Chemical tools

## Abstract

This paper examines the effects that electromagnetic fields from microwave radiation have in enzymatic reactions. Hydrolysis of proteins in beef (*in vivo* case) and casein (*in vitro* case) by the *papain* enzyme, a major industrial enzyme, is used herein as a model reaction to assess, under highly controlled conditions, the various parameters of microwave radiation (electric field, magnetic field, pulsed microwave irradiation, continuous microwave irradiation) as they might influence these *in vivo* and *in vitro* enzymatic reactions. The effect(s) of the microwaves’ electromagnetic fields was clearly evidenced in the *in vivo* case, contrary to the *in vitro* case where no such effect was observed, likely due to the nature of the hydrolysis reaction and to the autolysis (self-digestion) of the papain enzyme. Additionally, the effect of pulsed *versus* continuous microwave irradiation was further assessed by examining the *catalase*-assisted decomposition of hydrogen peroxide.

## Introduction

The interaction of electromagnetic fields with various life processes has intrigued scientists since the 1800s. Of current interest are the electromagnetic fields present in microwave radiation, which spans a frequency from 300 GHz to 300 MHz (i.e., from a wavelength of 1 mm to 1 m). Microwaves are used widely in communications and in heating, particularly in the heating of foodstuffs. This non-ionizing electromagnetic radiation is absorbed at the molecular level causing changes in the vibrational energy of the molecules; it also manifests itself as heat^1^. Identifying and evaluating biological effects of microwaves have been rather complex and controversial. In this regard, enzymatic reactions involving microwaves have been the object of active investigations in the latter half of the 20^th^ century, with the first such study reported in the early 1970s. For instance, microwave radiation to inactivate the enzymes in the brain of animals has been used widely since Stavinoha and coworkers first introduced it in 1970^2^. Inactivating the enzymes makes it possible to sample and measure many enzymatically destroyed brain neurochemicals thereby reducing the influence of postmortem changes^3^. Usage of microwaves has succeeded in stopping enzymatic activity selectively with minimal damage to other cells and/or proteins under ***in vivo*** conditions, for which equipment is commercially available.

Recent years have also witnessed investigations into the promoting effect of microwaves in enzymatic activity of ***in vitro*** systems at the molecular level^4^. In this regard, Young *et al*.^5^ reported that hyperthermophilic enzymes can be activated at temperatures far below their optimal temperature, presumably through a microwave-induced conformational flexibility. This finding offered the prospect of using hyperthermophilic enzymes at ambient temperatures to catalyze reactions with thermally labile substrates and products. Additionally, microwaves could be used to regulate biocatalytic rates of enzymes at very low temperatures from less thermophilic sources^5^. Parenthetically, Damm and coworkers^6^ critically evaluated microwave-assisted proteomic protocols. Upon examining microwave heating *versus* conventional heating, these authors^6^ asserted that non-thermal effects (i.e., microwave specific effects) had no influence on the structure and on the enzymatic digestion of proteins. Although there are various practical examples in the industry, discussion continues on the effects of the microwaves’ electromagnetic fields at the laboratory level.

Whether or not the microwaves’ electromagnetic fields affect enzymatic reactions necessitates the following basics to discuss appropriately this question: (i) no exchange of data exists between *in vivo* and *in vitro* cases, and (ii) while many of the discussions dealt with the nature of the samples, parameters pertaining to the microwave radiation have been largely ignored. For this reason, unless the effects of microwave radiation (e.g., ***electric field, magnetic field, continuous microwaves, pulsed microwaves***) and the like are examined, it is highly unlikely that the data can be reproduced whenever the apparatus is changed. In most studies, it was difficult to discuss microwave-induced phenomena because microwave devices with precise control of the microwaves were not used, even if specialized equipment for microwave chemistry were available^7^. Additionally, even if a commercially available chemical reactor were available, problems would have arisen nevertheless at the time because of the use of magnetrons^7^.

In our research strategy, we aimed to clarify the electromagnetic wave effect(s) of microwaves on enzymatic reactions through the use of a prototype device that could accurately control the incident microwaves, a device consisting of a semiconductor microwave generator, a power sensor and a fiber optic thermometer to measure temperatures in a single-mode applicator. In such a case, if a result appeared different from ordinary heating (acceleration or deceleration of the enzyme reaction), it would be a phenomenon attributable to an electromagnetic wave effect(s) unique to microwaves; obviously, a similar phenomenon would not be obtained with conventional heating. Furthermore, if the enzyme were selectively heated and exposed to a temperature field higher than in aqueous media, the rate of the enzymatic reaction would decrease, and in some cases the enzyme would be inactivated. With this strategy, the effects of the microwaves’ electromagnetic energy were examined from two different approaches: (1) the *in vivo* hydrolysis of the protein(s) in beef samples by the papain enzyme, and (2) the *in vitro* hydrolysis of the casein protein(s) by the papain enzyme used as a model reaction after being subjected to microwave radiation emitted from a semiconductor microwave generator. For comparison, reactions were also carried out under the more conventional heating methods (electric furnace and/or water bath). To the extent that the microwaves’ electromagnetic fields might affect directly only the enzyme molecule, we also examined the possible occurrence of the self-digestion of papain. The effects of pulsed-wave versus continuous microwave irradiation were also assessed by an examination of the decomposition of H_2_O_2_ with another enzyme: the ***catalase*** enzyme.

The papain enzyme, used herein as a model enzyme, is a cysteine protease enzyme largely used as a meat tenderizer via the breakdown of tough meat fibers, thereby rendering the meat easier to cook. This enzyme is used extensively in the textile, pharmaceutical, and cosmetic industries. The pharmaceutical applications of papain include tooth whitening, cleaning dead tissues in chronic wounds, and cell dissociation in cell culture techniques. The leather industry also uses papain for the tanning of leather. Increases in health awareness, rise in demands for meat tenderizers, and surge in demands for natural enzymes have driven the growth of the papain powder market^8^. Promoting the activity of this enzyme is a field of much attention. If the enzymatic activity of papain could be enhanced by the microwaves’ electromagnetic fields, it would lead to significant industrial benefits.

## Results and Discussion

### Evaluation of papain’s activity in an *in vivo* microwaved process

**Figure 1:**
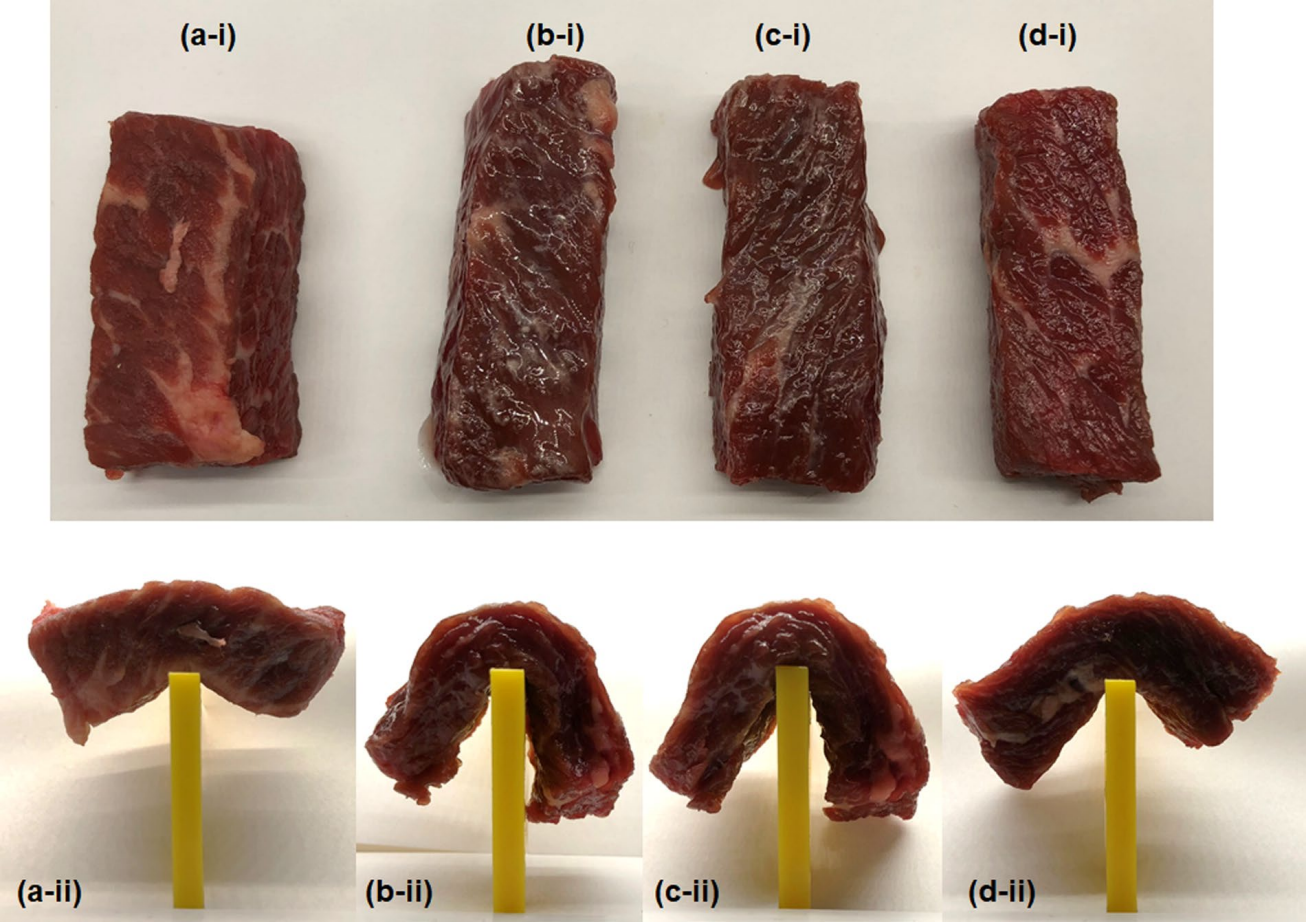
Observations of the surface changes (i) and the degree of softening (ii) of beef samples after applying papain to the beef: (**a**) control experiment with no papain used on the beef, (**b**) papain-treated beef sample after heating with pulsed microwave irradiation (PMI) and maintaining it at 45 °C for 10 min (**c**) papain-treated beef sample subjected to continuous microwave irradiation (CMI) under otherwise identical conditions as in (**b**), and (**d**) papain-treated beef sample subjected to heating in an electric furnace at 45 °C for 10 min.

Possible changes in the degree of enzymatic activity in the papain-assisted hydrolysis of proteins present in beef samples were examined by comparing microwave heating relative to conventional heating. The hydrolysis in beef samples whose surface was covered with the papain enzyme was used as the model of an *in vivo* process. Most of the beef samples were cut into 20 × 20 × 50 mm rectangularly-shaped specimens as illustrated in Fig. 1a-i,a-ii that display the initial papain-free beef sample. Changes in the condition of the surface subsequent to treatment of the beef samples with the papain enzyme (Fig. 1b-i,c-i,d-i) and the decomposition of the beef’s protein(s), as revealed by their response to gravity (Fig. 1b-ii,c-ii,d-ii), were examined in comparison with the untreated beef specimen of Fig. 1a that was subjected to the same gravitational condition.

The papain-treated beef samples were subjected to pulsed microwave irradiation (PMI; 20-millisecond pulses; Fig. 1b-i) and to continuous-wave microwave irradiation (CMI; Fig. 1c-i). Under microwave heating, the papain-treated samples of Fig. 1b-i,c-i softened in a manner suggestive of liquefied jelly when compared to the non-irradiated papain-free initial sample (Fig. 1a-i). That is, it shows that exposing the papain-coated beef specimens to microwave heating enhanced the decomposition of the protein(s) on the beef surface. We also observed that the degree of softness of the beef surface under PMI conditions was, to some extent, greater than under CMI. By contrast, upon being subjected to heating in an electric furnace at 45 °C for 10 min, the papain-treated beef sample of Fig. 1d-i softened much less than under microwave irradiation. Moreover, after the conventional heat treatment, the specimen’s surface was unlike the jelly-like surface displayed by the samples of Fig. 1b,c. Experiments were also conducted at 50 °C (+5 °C) and 40 °C (−5 °C) in the electric furnace to examine whether errors in temperature measurements could explain this difference. There were no differences at both these temperatures. Accordingly, despite the same temperature conditions (45 °C), the microwaves promoted the activity of papain on the beef surface that we attribute to the microwaves’ electromagnetic fields.

A closer look at the photographs in Fig. 1a-i,b-i,c-i,d-i shows that the sizes of the samples differed after their treatment. After microwave heating (Fig. 1b-i,c-i) and electric furnace heating (Fig. 1d-i), their longitudinal and lateral sizes were longer and narrower relative to the untreated beef specimen (Fig. 1a-i). The two beef specimens exposed to PMI and CMI irradiation were longer by about 2 cm, while the specimen heated in the electric furnace was ca. 1 cm longer than the untreated beef. These changes are due to the papain-assisted hydrolysis that cleaved the beef’s internal protein chains with the efficiency expected to increase under the microwaves’ electromagnetic fields relative to conventional heating. To confirm the cleavage of the internal protein chains, we examined the effect that gravity had on the heated and non-heated samples. To do so, we placed all four samples at the center of a vertical yellow plastic plate (5 mm thick and 10 mm wide) in a manner reminiscent of a seesaw.

The papain-free untreated beef sample remained firm to gravity as illustrated in Fig. 1a-ii; that is, there was no change in shape even though the sample was left standing for 5 min in this state. By comparison, it is clear from Fig. 1b-ii,c-ii that the papain-treated beef specimens subjected to microwave irradiation were unable to counter the gravity as they drooped down considerably compared to the untreated specimen of Fig. 1a-ii. It became also clear that they could not keep their initial flattened shape for more than 20 seconds, which confirms the cleavage of the protein chains inside the beef (softening) by the papain enzyme. A comparison of the extent of drooping in Fig. 1b-ii,c-ii shows that the hydrolysis of the beef protein was somewhat greater when the sample was subjected to PMI hydrolysis than with CMI. These experiments were repeated no less than four times, and in every instance the hydrolysis reaction was more pronounced under PMI than under CMI conditions. Upon being subjected to heating in an electric furnace at 45 °C for 10 min, the papain-treated beef sample softened somewhat, but significantly less than the microwaved specimens by the electromagnetic fields as evident from comparing the specimen of Fig. 1d–ii with the specimens of Fig. 1bc-ii after 5 min standing in that state.

The results afford two possible mechanistic considerations. In the first instance, the hydrolysis of the beef protein(s) by the papain enzyme was enhanced under PMI compared to CMI conditions, as the microwaves applied power for PMI was 1.8 times higher than that of CMI (11 W). Evidently, the microwave power level influences the papain’s activity and, not to be precluded, by the instantaneous electric field and magnetic field enhancement under microwave pulsed irradiation. In the second instance, microwave heating occurred first in the inner core of the beef samples, and to the extent that the surface temperature was that of the surrounding atmosphere, thus cooler than within the inner core, a temperature gradient was established under microwave heating^9^. Consequently, some of the moisture contained within the bulk moved to the surface, thus causing the enzymatic hydrolysis reaction to occur mostly at the specimens’ surface, as the extent of diffusion of the papain enzyme into the beef was limited. Thus, migration of the water in the bulk of the specimens to the surface had little consequence on any reaction occurring in the bulk. For the conventionally heated specimen, because the temperature inside the electric furnace was high, any moisture on the surface of the beef sample tended to evaporate, thereby causing the hydrolysis reaction at the surface to be inhibited, as evidenced by the results shown in Fig. 1d-ii relative to Fig. 1bc-ii. In summary, compared to conventional heating, microwave heating presents certain features that promote the activity of the papain enzyme in the *in vivo* case that we attribute to the accelerating effect of the electromagnetic fields of the microwave radiation.

In other experiments we observed that after applying the papain enzyme to the surface of the beef samples, and then allowing them to stand at ambient temperature for 10 min; they displayed nearly the same features as the specimen used in the electric furnace heating experiment. On the other hand, as shown in Fig. 1b,c, microwave-irradiated beef not coated with papain displayed no observable softening. Therefore, it was expected that the microwaves alone were not capable of softening the beef. In addition to these confirmatory experiments, we also conducted similar experiments on beef sold at supermarkets (Angus beef; imported from the USA). Results were similar to the trends illustrated in Fig. 1. Softening of beef specimens accompanying the enhancement of the activity of the papain enzyme by microwaves does not appear to be a phenomenon caused by the state of beef.

### Evaluation of papain’s activity in an *in vitro* microwave-assisted enzymatic reaction

To evaluate the possible microwaves’ electromagnetic field effects in an *in vitro* case, we examined the hydrolysis of ***casein*** (a model protein) in the presence of the papain enzyme performed with microwave elements otherwise the same as those used in the previous *in vivo* system. Casein consists of a family of related phosphoproteins (αS1, αS2, β, κ) that is commonly found in mammalian milk. It contains a high number of non-interacting proline residues and no disulfide bridges so that it has relatively little tertiary structure; it is relatively hydrophobic making it poorly soluble in water, although it can be dispersed in dilute alkaline media and in salt solutions.

**Figure 2:**
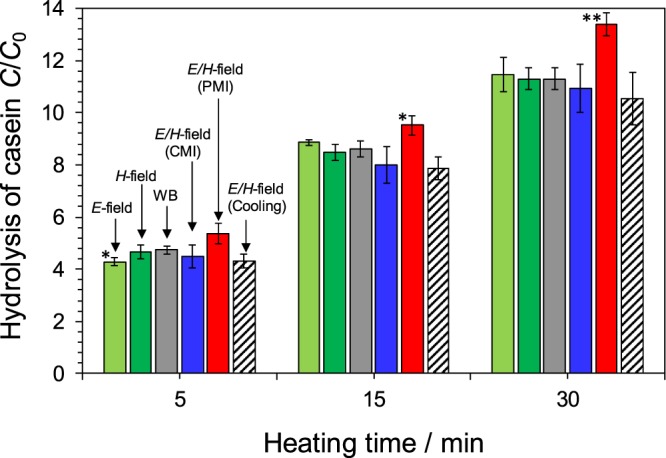
Hydrolysis of casein by the papain enzyme monitored by the UV absorption at 275 nm after 5, 15 and 30 min heating (*C*) against 0 min (*C*_0_); the reactor was located at the maximal position of the electric field density (*E*-field), at the maximal position of the magnetic field density (*H*-field), by water-bath heating (WB), by electric-/magnetic-field heating (*E/H*-field) under continuous microwave (CMI) or pulsed microwave (PMI; 20-millisecond pulse) irradiation, and by *E/H*-field heating under cooling conditions (*E/H*-field-cooling); temperature was in all cases 60 °C. Note that * and **: Student’s t-test significant at p < 0.05 and p < 0.01, respectively, compared to WB.

The increase in the efficiency of the papain-assisted hydrolysis of casein was followed by monitoring the UV absorption band at 275 nm after 5, 15 and 30 min heating against 0 min (Fig. 2) for each heating method, namely (a) *E*-field and *H*-field microwave heating, (b) water-bath heating (WB), (c) *E/H*-field heating under continuous microwave irradiation (CMI) and pulsed microwave irradiation (PMI; 20-millisecond pulses), and (d) *E/H*-field heating under cooling conditions (*E/H*-field-cooling) at 60 °C.

In the first case, we looked at the effects that the microwaves electric-field (*E*-field) and magnetic-field (*H*-field) heating might have on such an *in vitro* process. As evident in Fig. 2, there was no significant difference in the rate of hydrolysis of casein at all three heating times by *E*-field heating, *H*-field heating and by water-bath heating. The only significant difference by the t-test was 5 min of *E*-field. (see Table S2 in the Supplementary Information). Moreover, within experimental error, no enhancement of the hydrolysis of casein was seen in the presence of the papain enzyme under microwave and conventional heating. In addition, a comparison between *E*/*H*-field (CMI) and *E*/*H*-field (PMI) conditions, as done for the *in vivo* case, shows that the efficiency of the hydrolytic process is greater under PMI than WB heating. However, the efficiency of the papain enzymatic reaction under PMI conditions did not increase dramatically (The only significant difference by the t-test was 15 and 30 min of *E*/*H*-field). Under CMI conditions the efficiency was below that of WB heating.

The hydrolysis of casein by the papain enzyme, carried out under microwave heating while cooling the solution using the GPC-1000 microwave chemical equipment with an aluminum cooling block, permitted using more microwave input power (+3 Watts) to irradiate the solution while keeping the temperature constant at 60 °C. Under the latter conditions, no change in casein hydrolysis efficiency was observed even when the additional 3-Watt microwaves were used to irradiate while cooling the sample. In all microwave-heating instances, it is somewhat curious that the effects of the microwaves’ electromagnetic fields did not manifest themselves more clearly in the *in vitro* reactions.

**Figure 3:**
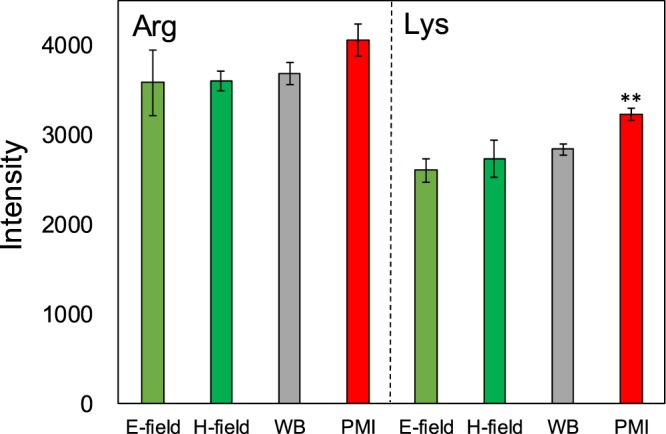
Increase in the intensity of the fluorescence spectral band at 393.8 nm following the papain- assisted hydrolysis of **Arg**: *t*-butyloxycarbonyl-L-phenylalanyl-L-seryl-L-arginine-4-methylcoumaryl-7-amide (Boc-Phe- Ser-Arg-MCA) and **Lys**: *t*-butyloxycarbonyl-L-valyl-L-leucyl-L-lysine 4-methylcoumaryl-7-amide (Boc-Val-Leu- Lys-MCA) after 2 min by the microwaves’ electric field heating (*E*-field), magnetic field heating (*H*-field), water-bath heating (WB), and pulsed microwave irradiation (PMI; 20-millisecond pulses). Note that * and **: Student’s t-test significant at p < 0.05 and p < 0.01, respectively, compared to WB.

As the efficiency of the hydrolysis of casein by the papain enzyme displayed little if any changes under both microwave heating and conventional heating, the focus next shifted to investigate whether the effect of the microwaves’ electromagnetic fields could be observed in the papain-assisted hydrolysis of substrates other than casein. Accordingly, we examined the fate of the amino acid trimers *t*-butyloxycarbonyl-L-phenylalanyl-L-seryl-L-arginine-4-methyl-coumaryl-7-amide (Boc-Phe-Ser-Arg- MCA) and the *t*-butyloxycarbonyl-L-valyl-L-leucyl-L-lysine-4-methylcoumaryl-7-amide (Boc-Val- Leu-Lys-MCA). The papain enzyme preferentially cleaves the bond between the Arg and MCA entities and between the Lys and MCA entities. Results of the hydrolysis of the amino acid trimers in aqueous media containing the papain enzyme using four heating methods are displayed in Fig. 3. Beyond experimental errors and except perhaps the PMI condition for Lys, there were no dramatic differences between the heating methods employed on the efficiency of the hydrolysis reaction of the two amino acid trimers.

### Possible reasons why the microwaves’ electromagnetic field effects are not evident in the *in vitro* enzymatic reaction

Coating beef samples with the papain enzyme (*in vivo* case) clearly promoted the hydrolysis of the meat protein(s) by microwave heating. However, in the *in vitro* case, a similar outcome was not seen in the hydrolysis of the casein protein. A comparison between *in vivo* and *in vitro* conditions shows a non-insignificant gap of reaction conditions. Germane to the present study, our previous study^10^ may prove helpful in understanding the lack of influence of the microwave radiation on the hydrolysis of casein by the papain enzyme. In that study, we reported on the acid hydrolysis of cellulose to produce glucose in the presence of AC-SO_3_H catalyst particles (AC, activated carbon) subjected to microwave heating and conventional heating. Results showed that microwave heating promoted the selective overheating of the AC-SO_3_H catalyst and thus the rapid hydrolysis of cellulose was expected under microwave selective heating of the catalyst. However, even though selective heating of the catalyst occurred, we found no difference in reaction efficiency in the acid-catalysed acid hydrolysis of cellulose whether the reactants were heated by microwave heating or by conventional heating. We concluded that the hydrolysis of cellulose did not take place near the catalyst active site, but rather occurred in the solution bulk such that the effect of the microwaves was diminished. Following this reasoning, in the *in vitro* papain-assisted hydrolysis of the casein protein, the enzyme acted merely as a trigger for the hydrolysis. The process occurred in aqueous media with the rate-limiting factor being the heat in the aqueous solution, so that the microwaves’ electromagnetic field effects appear relatively inconsequential, although the results did not preclude a slight pulsed microwave effect.

### Influence of the self-digestion (autolysis) of the papain enzyme during the hydrolysis

Other than heat, do microwaves have any other influence on enzymatic reactions? To resolve this issue, we next focused on events that may occur within the enzyme itself. Protease enzymes such as papain typically hydrolyze proteins, yet at the same time papain seems to be an atypical enzyme as it may undergo self-digestion (autolysis) as evidenced by the quantity of enzyme remaining in solution that decreased with reaction time.

**Figure 4:**
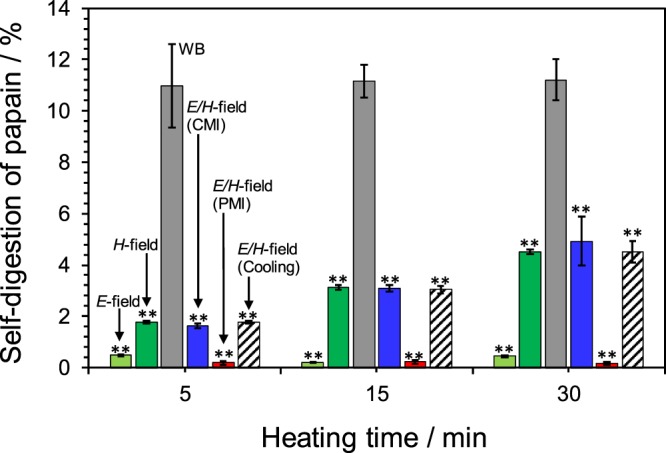
Percent self-digestion of papain monitored by the UV absorption at 245 nm for 5, 15 and 30 min heating against 0 min; the reactor was located at the maximal position of the electric field density (*E*-field), at the maximal position of the magnetic field density (*H*-field), by water-bath heating (WB), by electric/magnetic-field heating (*E/H*-field) under continuous microwave (CMI) or pulsed microwave (PMI; 20-millisecond pulses) irradiation, and under *E/H*-field heating with cooling conditions (Cooling) at 60 °C. Note that * and **: Student’s t-test significant at p < 0.05 and p < 0.01, respectively, compared to WB.

The heating of aqueous solutions containing only the papain enzyme (without the casein) at 60 °C was carried out using each of the heat sources reported in Fig. 4, which also displays the extent of self-digestion of the enzyme monitored by absorption spectra recorded at 0, 5, 15 and 30 min. A comparison between the microwaves’ *E*-field and *H*-field heating reveals that the self-digestion of the enzyme is suppressed under *E*-field heating; in fact, changes in absorption in the spectra were hardly observed under the latter condition. Under *H*-field heating conditions, however, the extent of self-digestion increased with heating time. Most significant, under water-bath heating, the extent of self-digestion of papain was significantly greater vis-à-vis microwave heating and remained unchanged with heating time.

Results from the self-digestion experiments of aqueous solutions of papain (no casein) subjected to microwave *E*/*H*-field irradiation under CMI and PMI conditions showed that the self-digestion of this enzyme under PMI was significantly inhibited relative to CMI. Since an apparent pulsed microwave effect was observed at 60 °C, we consider that suppression of the self-digestion of papain was affected by an electromagnetic field (In all experimental data, the t test showed a significant difference of less than 0.01). Performing microwave heating while cooling using the GPC-1000 microwave chemical equipment with an aluminum cooling block, more microwave input power (+3 Watts) was used in causing the self-digestion of papain also at 60 °C. Yet even with cooling, inhibition of the self-digestion of papain was remarkably absent compared with *E/H* fields with CMI. For the *in vitro* case, although the microwaves’ electromagnetic fields had no influence in the hydrolysis of casein by the papain enzyme, they likely did play a role in the attenuation/inhibition of the self-digestion of this protease enzyme. This can be taken to mean that the self-digestion of papain is not an aqueous media event, but rather it is an intramolecular event within the enzyme. More importantly, the effect of pulsed microwave radiation was significant as demonstrated in Fig. 4.

### Effect of pulsed microwave irradiation on the *catalase*-assisted degradation of hydrogen peroxide

The issue of whether or not there is an effect of the microwaves’ electromagnetic fields in enzymatic reactions was examined using three different approaches: (i) the extent of softening of beef specimens coated with the papain enzyme (*in vivo* case); (ii) the hydrolysis of casein in the presence of papain in homogeneous aqueous media (*in vitro* case); and (iii) the self-digestion of papain (*in vitro* case). All these approaches revealed a commonality, namely the effect of pulsed microwave irradiation (PMI).

**Figure 5:**
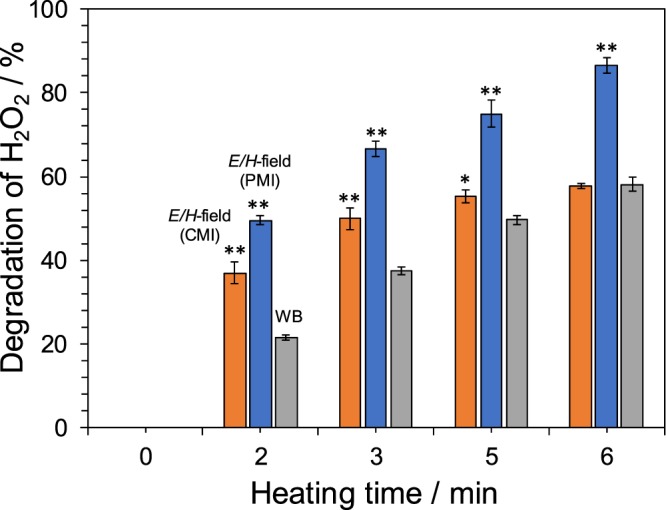
Residual quantity of H_2_O_2_ in the catalase-assisted degradation of hydrogen peroxide under microwave continuous irradiation (CMI), pulsed microwave irradiation (PMI), and conventional heating with a water bath (WB). Note that * and **: Student’s t-test significant at p < 0.05 and p < 0.01, respectively, compared to WB.

In a previous study^11^, we demonstrated the advantage of using microwave heating against water-bath heating in the decomposition of hydrogen peroxide (2H_2_O_2_ → 2H_2_O + O_2_) in the presence of the metalloenzyme ***catalase***. Consequently, we elected to investigate also the effects of microwave radiation pulses using this catalase-assisted reaction. Results displayed in Fig. 5 clearly demonstrate that pulsed microwave irradiation promoted the activity of the catalase enzyme. On the other hand, in CMI, the decomposition rate decreased after 3 min. Our previous study (using CMI) showed that using microwaves in catalase reaction can only accelerate the initial degradation rate^11^. This clarified that local heating in the enzyme molecule was increased by the effect of the microwaves, thereby causing the deactivation of the enzyme. To confirm this hypothesis, we further clarified that by allowing the enzyme to react at 25 °C (optimal reaction temperature: 37 °C), the reaction promoting effect by the microwaves was maintained^11^. Partial heating field can be suppressed by using PMI because the cooling of the enzyme progresses when the microwaves are turned OFF with respect to microwave heating. As a result, the promoting effect of the enzyme reaction by the microwaves was sustained.

Although not well documented, microwave apparatuses for chemical syntheses that utilize a microwave oven or an AC transformer power source emit microsecond-level pulses, even though the microwaves oscillate continuously. In our previous studies, many of the organic chemical reactions could not benefit from the microwaves’ electromagnetic fields because the reactions were driven mostly by thermal energy^7^. On the other hand, in photocatalytic organic synthesis reactions that involved Pt as the catalyst, the pulsed microwave effect was clearly demonstrated^7^. In enzymatic reactions, however, the existence of an effect of pulsed microwaves’ electromagnetic fields seems to depend on the nature of the reaction. Therefore, the dependence on the microwave apparatus is likely to be a relevant factor in taking full advantage of the microwaves’ electromagnetic fields in enzymatic reactions.

## Final Remarks

In the *in vivo* case, wherein the papain enzyme was directly adsorbed/absorbed on the surface of the beef samples, the enzyme-assisted hydrolysis of the beef protein(s) is a solid/solid reaction. As such, the influence of the microwaves on the hydrolytic process by the papain enzyme was accentuated relative to the case where the hydrolysis might occur by heating the liquid medium. No doubt, the microwaves stimulated the interactions between the papain enzyme and the meat protein(s). These results are quite instructive. When carrying out industrial applications of microwave radiation, an initial examination of the reaction system may prove useful in establishing whether the process is effective or is otherwise influenced by the microwaves. Nonetheless, in real complex reaction systems, unexpected different useful results may well appear. Since no temperature measurements at the molecular level have been done in this experiment, it was not possible to discuss whether this is a thermal effect or a non-thermal effect. However, there is no doubt that it is an electromagnetic wave effect unique to microwaves. By contrast, there was no advantage of using microwave irradiation to enhance the hydrolysis activity of papain in the *in vitro* systems, because the hydrolysis that occurred in homogeneous aqueous media was driven thermally, so that the effect of the microwaves was rather small. However, the promoting effect of microwaves was clearly demonstrated in the intramolecular reaction of the self-digestion of papain. The effect of 20-millisecond microwave pulses was clear. Generally, pulsed-wave and continuous-wave conditions are determined by the specifications of the power supply of the microwave device. From the points that do not appear in all reactions or appear in pulses, there is nonetheless room for discussion of the possibility that non-thermal effects due to microwaves may be involved. Consequently, if one pays no attention to this point, even if the reactions were carried out at the same temperature, the maximum amount of microwave energy would vary greatly, which could lead to issues of reproducibility in the experiments.

For the electromagnetic wave effect(s), microwave heating progresses directly through absorption of the microwave energy by molecules and ions. The energy transmission and heat generation efficiency are significantly higher than through conventional heat transfer. This can be associated to the fact that microwave heating rate is faster than traditional heating. This fact reminds us of the following: despite the same thermal energy state, expansion of the entropy of molecules and clusters associated with heat production is expected to be slow. If this difference contributed to the reaction, it would either promote or reduce reaction efficiency. In the near future and with additional studies, it may be possible to purposely control the effect(s) of electromagnetic waves in enzymatic reactions.

## Experimental

### Microwave apparatus

**Figure 6:**
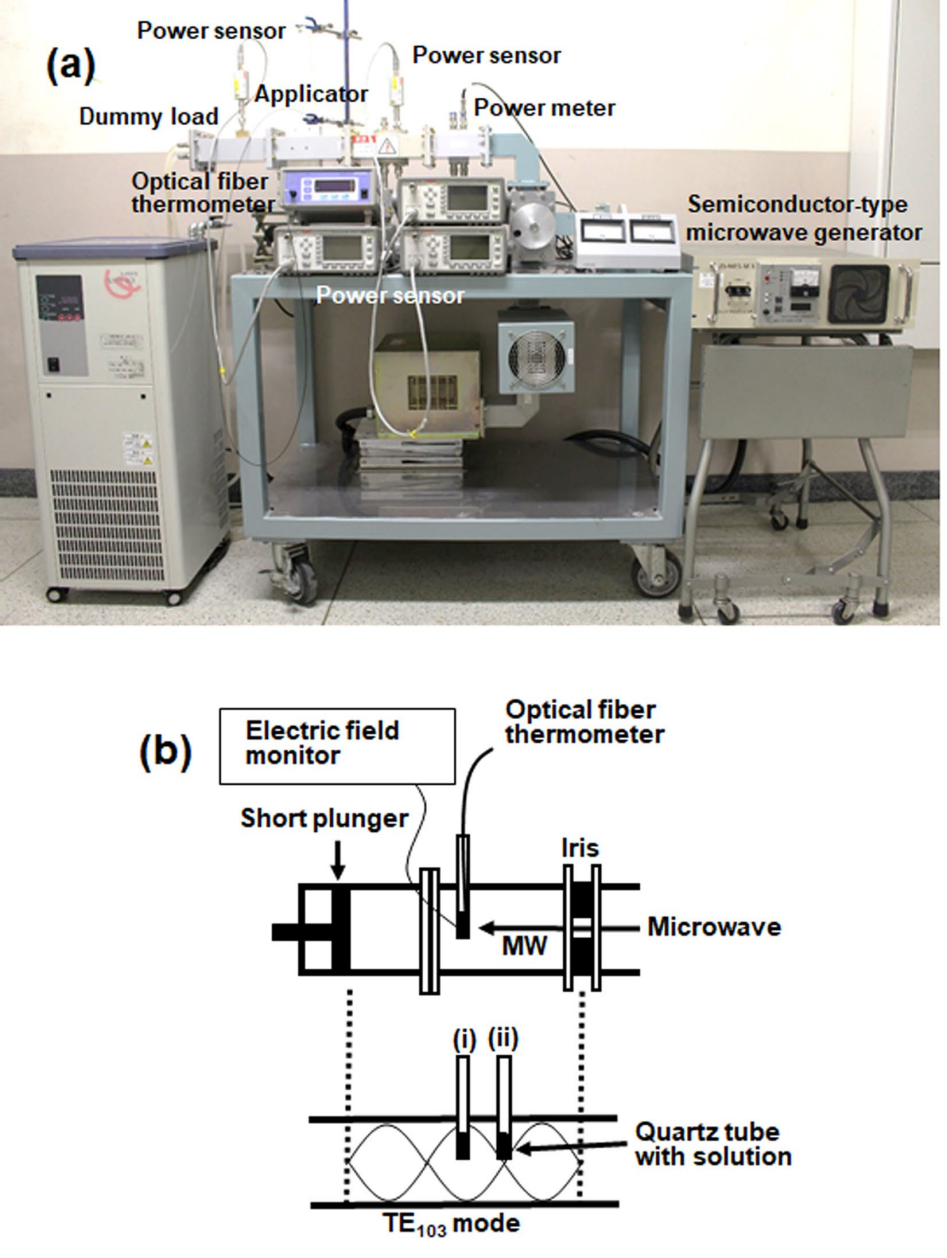
(**a**) Photograph of the microwave heating system using a microwave semiconductor generator, a single channel power meter and a power sensor, isolator, single-mode applicator and a dummy load. (**b**) detailed illustration of the experimental setup and positioning of the samples in the single-mode TE_103_ cavity; (i) maximal position of the electric field (*E*-field) density; (ii) maximal position of the magnetic field (*H*-field) density.

The schematics and an actual photograph of the microwave device used in the experiments are illustrated in Fig. 6. The microwave generator utilized a semiconductor-type microwave generator system {SPC Electronics Corp.: maximum output power, 240 W}. A generator was connected to a waveguide (WRJ-2), which was connected with a coaxial waveguide converter to the generator and to the sample heating applicator through an isolator, and to the incident and reflection sensors. A water-cooled dummy load was connected to the tip of the applicator (for *E/H*-field-CMI and *E/H*-field-PMI methods); the sample was subsequently irradiated with a traveling wave without resonating the microwaves. The CMI and PMI were obtained from a semiconductor microwave generator; under PMI conditions, the microwaves irradiated the sample(s) for 20 milliseconds (50 Hz duty cycle) and then turned OFF for 20 milliseconds. This pulse condition is the pulse width generated by most of magnetrons driven by a 50 Hz power supply as commercial power frequency. That is, it imitates the oscillation of most microwave ovens and industrial microwave irradiation equipment. The incident microwaves were absorbed with a dummy load; under the conditions used, none of the microwaves were reflected. The reason for this was to prevent non-uniform temperatures at the samples owing to unevenness of the electromagnetic wave at the samples’ position originating from resonance conditions. Also, a power sensor was connected between the sample and the dummy load to monitor the power of the microwave passing through it. On the other hand, in the experiment at the maximum electric or magnetic field, consisting of a single-mode TE103 cavity (transverse electric 103 mode) schematically illustrated in Fig. 6b, also included a short plunger, an iris, a three-stub tuner, power sensors and an isolator. The resonance of the microwaves was adjusted with the iris and the plunger at 1.5 cycles.

Heating the enzyme solution was achieved by locating the quartz tube reactor in the single-mode microwave apparatus (Fig. 6b) within the waveguide at positions either of maximal electric field (position (i)) or magnetic field (position (ii)) density. The maximal position of the *E* field from the iris was located at 3/4 the wavelength of the standing wave in the waveguide (i.e., at 11.09 cm)^12^. An electric field monitor (Fuji Electronic Industrial Co. Ltd.) was used to maintain the sample tube at the maximal position of the *E*-field density, as the reproducibility of such experiments is often diminished if such operations were neglected. Under our conditions, no significant positional changes of the electric field were necessary. The experiment of conventional heating was conducted using a water bath with stirrer (AS ONE Co. WBS-80M); the temperature of water in the water bath was fixed at 60 °C. Experiments for microwave heating and water-bath heating were repeated no less than six times; the average of the data is reported. Analogous conditions of thermometer, reactor, and the same solution prevailed under both conventional and microwave heating. The sample solution was contained in a branched test tube made of quartz (inner diameter, 12 mm); a reflux tube was connected to the top of the test tube; the temperature was measured using a fiber optic thermometer (Anritsu Meter Co., Ltd.). In preliminary experiments, we confirmed that the difference in temperature with respect to the vertical direction of the solution due to microwave irradiation was less than 1 °C. We also confirmed that during the experiments the reflection of microwaves was zero according to the power sensor.

**Figure 7:**
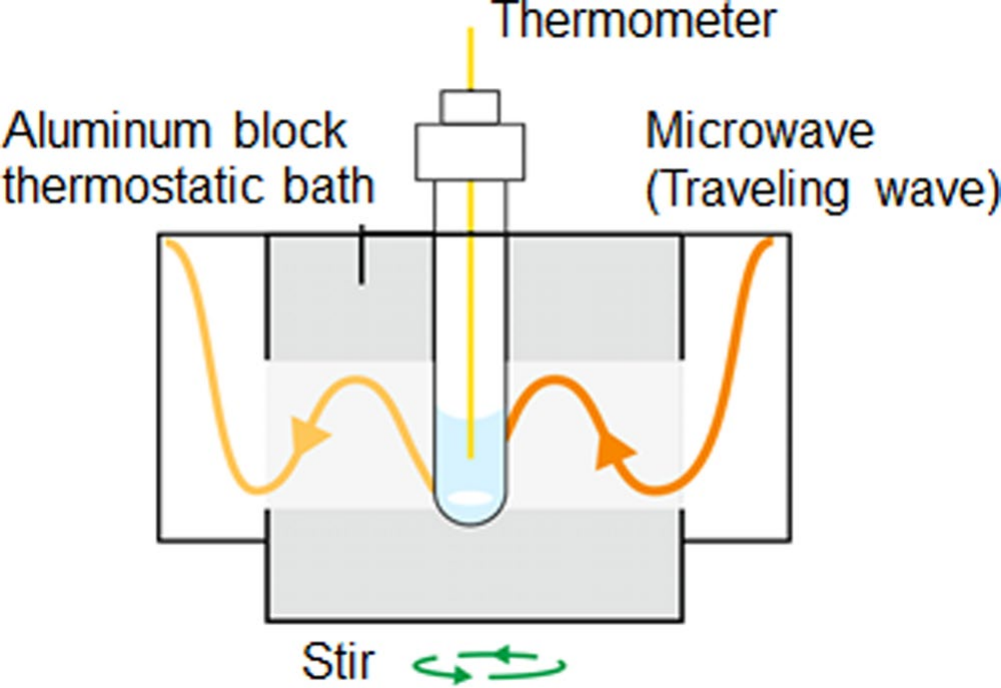
Cavity image diagram for heating with microwaves while cooling the sample with an aluminium block.

Microwave heating with a traveling wave (*E*/*H*-fields) under cooling conditions was also carried out using a GPS-1000 microwave chemical synthesis equipment (Tokyo Rikakikai Co. Ltd.). The Pyrex glass reaction vessel placed in the cooled thermostatic chamber aluminum block within the GPS-1000 system can be irradiated continuously with the traveling waves of the microwave radiation emitted from the semiconductor generator. The advantage of this apparatus is that it can be used to irradiate with microwaves while cooling as illustrated in Fig. 7. When the sample was irradiated at a microwave input power of 10 Watts, the temperature of the sample solution reached 60 °C within 5 min. By contrast, when the aluminum block was cooled to 30 °C, for the temperature of the solution to reach 60 °C within 5 min necessitated irradiation with 13-Watt microwave radiation; thus, cooling permitted the use of an extra 3 Watts of microwave power.

### Methodology for *in vivo* enzymatic reactions

In a practical *in vivo* experiment, a 10-mg powdered sample of the papain enzyme (Wako Pure Chemical Industries) was used to cover the whole surface of pieces of beef (dimensions ca. 20 × 20 × 50 mm). Microwave radiation was subsequently used to heat the pieces of beef with 11-Watt (continuous wave) and 20-Watt (pulse wave) microwaves in a system consisting of a single-mode microwave applicator (traveling microwave wave) that was kept at a temperature of 45 °C for 10 min. In pulsed microwave heating, the microwaves emitted from a semiconductor microwave generator were turned ON and OFF every 20 milliseconds. For comparison, the beef/papain samples were also heated and kept at ca. 45 °C for 10 min in an electric furnace previously brought to this temperature. In electric furnace heating, the temperature of the beef specimens was controlled and the potential overshoot of the temperature beyond 45 °C was prevented by opening and closing the door of the electric furnace. The temperature of all the beef/papain samples was measured at locations 3 mm below the surface of the beef sample using a fiber optic thermometer.

### Methodology for *in vitro* enzymatic reactions

For the *in vitro* experiments involving the papain enzyme and the casein protein, the enzyme (1.0 mg) was first added to ion-exchanged water (20.0 mL), whereas the casein (500 mg; Wako Pure Chemical Industries) was dissolved in a tris-HCl aqueous solution (0.10 M; 50.0 mL; pH 8.0). An enzyme activating solution (pH 8.0) was added to promote the activity of papain, which consisted of L-cysteine hydrochloride monohydrate (440 mg; Wako Pure Chemical Industries, Ltd.) and ethylenediamine-*N*,*N*,*N*′,*N*′- tetraacetic acid disodium salt dihydrate (0.27 g; Wako Pure Chemical Industries, Ltd.) dissolved in ion-exchanged water (45.0 mL). Subsequently, the casein solution (2 mL), the tris-HCl solution (0.010 M; 200 μL), the ion-exchanged water (200 μL), and the papain aqueous solution (600 μL) were added in this order to a quartz test tube reactor. The resulting solution was stirred using a micro-stirring bar and then subjected to a 4-Watt microwave electric field heating (*E*-field), to a 4-Watt magnetic field heating (*H*-field), and by conventional heating using a water bath. The initial temperature of the solution was 20 °C, after which the temperature of the solution was raised in each case to the reaction temperature of 60 °C (the heating rate was ca. 0.57 °C s^−1^). At these microwave input power levels, microwave heating and heat loss to the atmosphere were in equilibrium at 60 °C; no further temperature rise of the solution occurred even if subjected to further microwave irradiation. Preliminary experiments had shown that the hydrolysis of casein by the papain enzyme was no longer promoted at times over 30 min. Accordingly, heating times with microwaves and water-bath heating periods were fixed at 5, 15 and 30 min, following which an aqueous solution of trichloroacetic acid (5 w/v%; 3 mL) was added to terminate the reaction of papain. The denatured protein was precipitated by centrifugation (300 rpm) for 5 min, following which the aqueous solution was filtered and then analyzed spectroscopically with a UV/Vis spectrophotometer (JASCO V-760).

The hydrolysis of the amino acid trimers, *t*-butyloxycarbonyl-L-phenylalanyl-L-seryl-L- arginine-4-methylcoumaryl-7-amide (Boc-Phe-Ser-Arg-MCA; 0.01 mg) and of the *t*-butyloxy- carbonyl-L-valyl-L-leucyl-L-lysine-4-methylcoumaryl-7-amide system (Boc-Val-Leu-Lys-MCA; 0.01 mg) in the presence of the papain enzyme (1.0 mg) was carried out by addition of the amino acid trimers into 10 L of ion-exchanged water. In the decomposition of the amino acid trimer by the papain enzyme, the enzyme preferentially disconnects the arginine (Arg) terminal or lysine (Lys) terminal amino acid. In essence, following the hydrolysis at the Arg-MCA or Lys-MCA positions, the MCA is isolated causing it to fluoresce. The emission by the MCA was recorded at 393.8 nm using the JASCO FP-8000 fluorescence spectrophotometer; the excitation wavelength was 290 nm.

The enzymatic activity of catalase (from bovine liver; Wako Pure Chemical Industries, Ltd.) in the degradation of H_2_O_2_ in aqueous media was investigated by introducing 2 mg of catalase in an aqueous Na_3_PO_4_ buffered solution (50 mM; 15 mL). Three microliters (3 µL) of this catalase solution were subsequently added to a quartz tube reactor (dia., 12 mm; internal dia., 10 mm; height, 100 mm) followed by addition of 30 mL of an aqueous Na_3_PO_4_ buffered solution (50 mM; pH 8.0; 29.8 mL) containing 0.2 mL of 30% H_2_O_2_ (quantity of H_2_O_2_ in the resulting solution was 0.2%). The reactor containing a magnetic stirring bar (25 mm) was then positioned in the microwave equipment (microwave heating) or in a water bath (conventional heating). After a given reaction time (2, 3, 5 or 6 min), the solution was immediately put into boiling water bath for 1 min to arrest the activity of the enzyme. The quantity of H_2_O_2_ remaining in solution was monitored at 190 nm with a UV/Vis spectrophotometer (JASCO V-760). Analogous conditions of thermometer, reactor, and the same solution prevailed under both conventional and microwave heating.

### Statistical analysis

Statistical analyses were performed with 1 tailed and unpaired Student’s t-test using the formula eq. 1 (*SD*: standard deviation, *n* = number of samples, *i* = Water bath, *ii* = Microwave). Results from the calculations (Excel software) are indicated with average and standard deviation.

1 $${\rm{t}}=\frac{|Averag{e}_{i}-Averag{e}_{ii}|}{\sqrt{\frac{{(S{D}_{i})}^{2}}{{n}_{i}}+\frac{{(S{D}_{ii})}^{2}}{{n}_{ii}}}}$$

## Supplementary information

Supplementary information
