# Supplementary material for: Probing the effect(s) of the microwaves’ electromagnetic fields in enzymatic reactions
Source: Sci Rep. 2019 Jun 20;9:8945. doi: 10.1038/s41598-019-45152-9 (PMC6586677; doi:10.1038/s41598-019-45152-9)
Supplement: Supplementary file 1 — Supplementary information [file 41598_2019_45152_MOESM1_ESM.docx]

**Probing the effect(s) of the microwaves’ electromagnetic fields in enzymatic reactions**

**Satoshi Horikoshi, Kota Nakamura, Mikio Yashiro, Kanae Kadomatsu, and Nick Serpone**

**Supplementary Information**


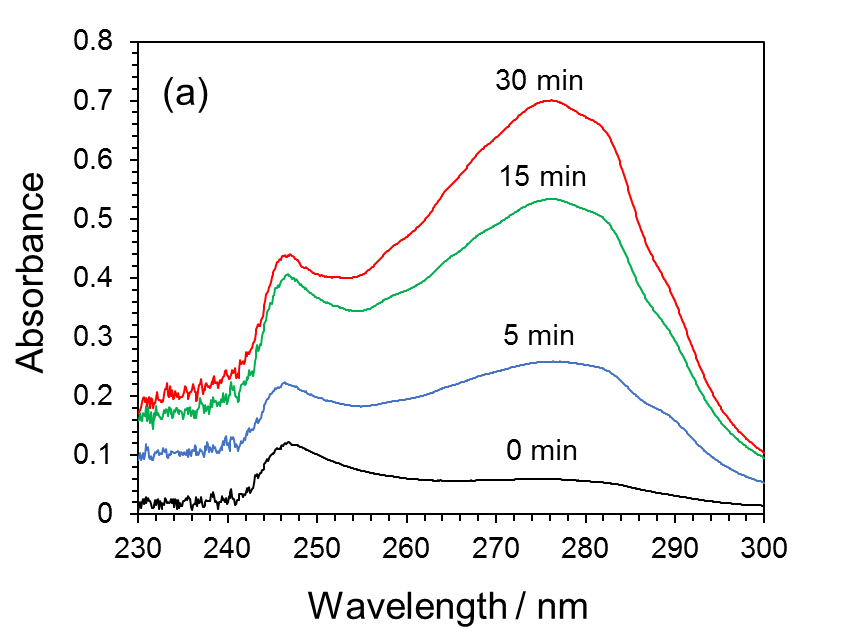

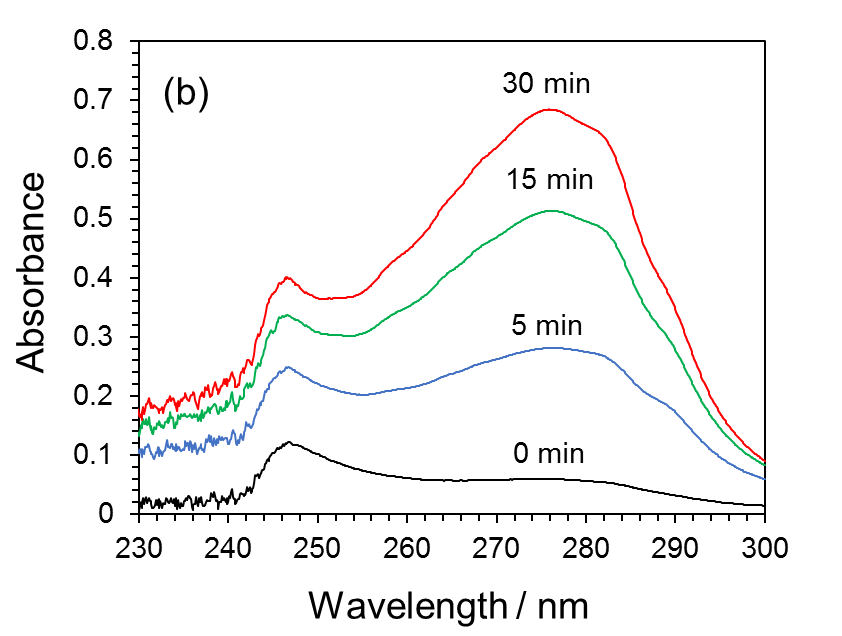


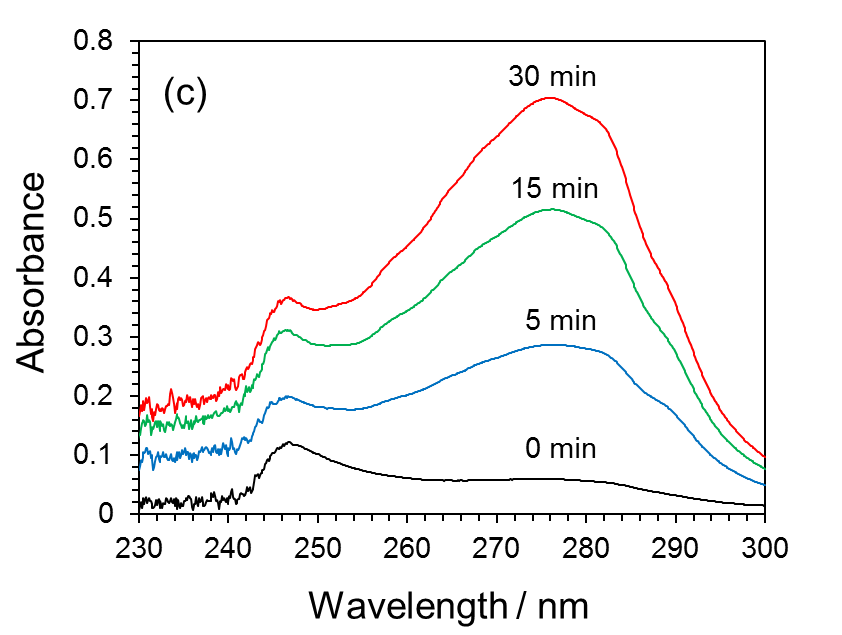


**Figure S1.** Time changes in the UV spectra following the hydrolysis of casein by the papain enzyme at 60 °C under conditions of heating the solution located (a) at the maximal position of the electric field density (*E*-field), (b) at the maximal position of the magnetic field density (*H*-field), and (c) by water bath heating (WB); (d) increase in UV absorption at 275 nm after 5, 15 and 30 min of heating relative to 0 min.

As an added characteristic, we also recorded the mass spectra of the samples in **Figure S2** in order to understand the lack of papain activity under microwave irradiation. The hydrolysis products from the casein solution after the hydrolysis for 15 min were analysed by the MALDI-TOFMS technique. The mass spectrum taken immediately after introducing papain into the casein solution and deactivated by addition of trichloroacetic acid (5 w/v%; 3 mL) is shown in **Figure S2a**, which displays a relatively strong peak (intensity, 170 a.u.) at m/z = 2623. Note that unless noted otherwise the temperature was maintained at 60 °C. By comparison, the mass spectrum recorded for the casein/papain sample solution heated for 15 min under microwave electric field (*E*-field) heating displayed (**Fig. S2b**) a notably lesser intense peak (ca. 55 a.u.) also at m/z = 2623. Under microwave magnetic field (*H*-field) heating for 15 min, the mass spectrum of the sample solution (**Fig. S2c**) also exhibited a lesser intense peak (ca. 55 a.u.) at m/z = 2623. By contrast, under the more conventional water bath heating for 15 min the mass spectrum (**Fig. S2d**) showed a peak at m/z = 2363 albeit more intense (ca. 190 a.u.) than under either *E*-field or *H*-field heating. The nature of the molecular species with m/z = 2623 remains elusive for the moment, but we hypothesize that the mechanism of casein decomposition assisted by the presence of the papain enzyme under microwave *E*-field or *H*-field heating may be significantly different from heating with the water bath method. Although we used an amino acid sequencer for the samples, we were unable to deduce the details of the species displaying those peaks. Nonetheless, there is no doubt that some different process is taking place in the hydrolysis of casein by the papain enzyme when using microwave heating.


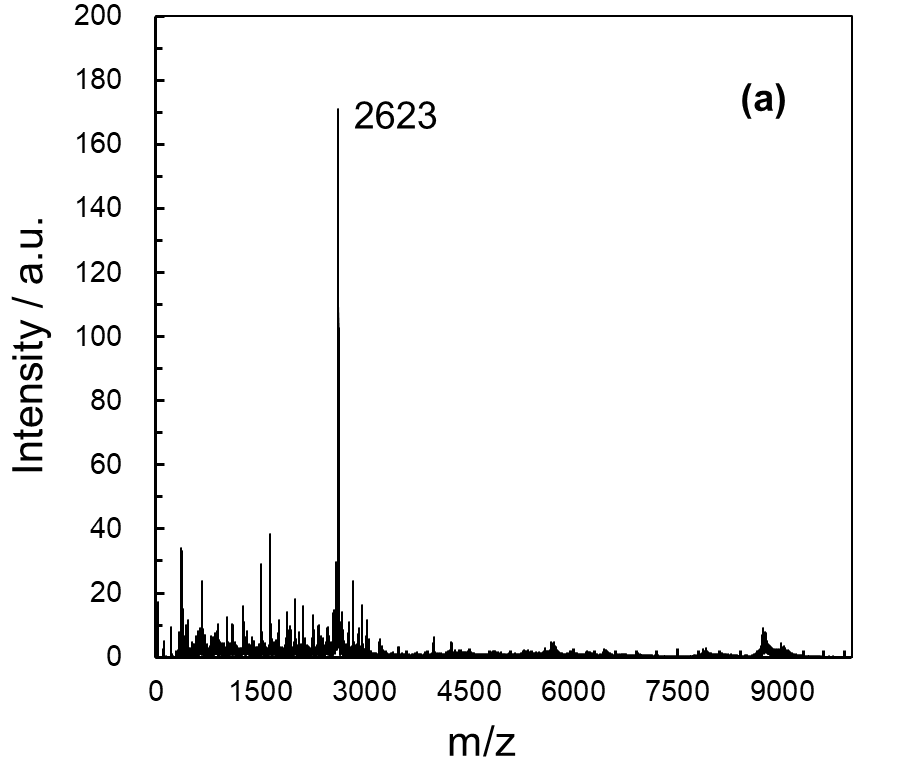

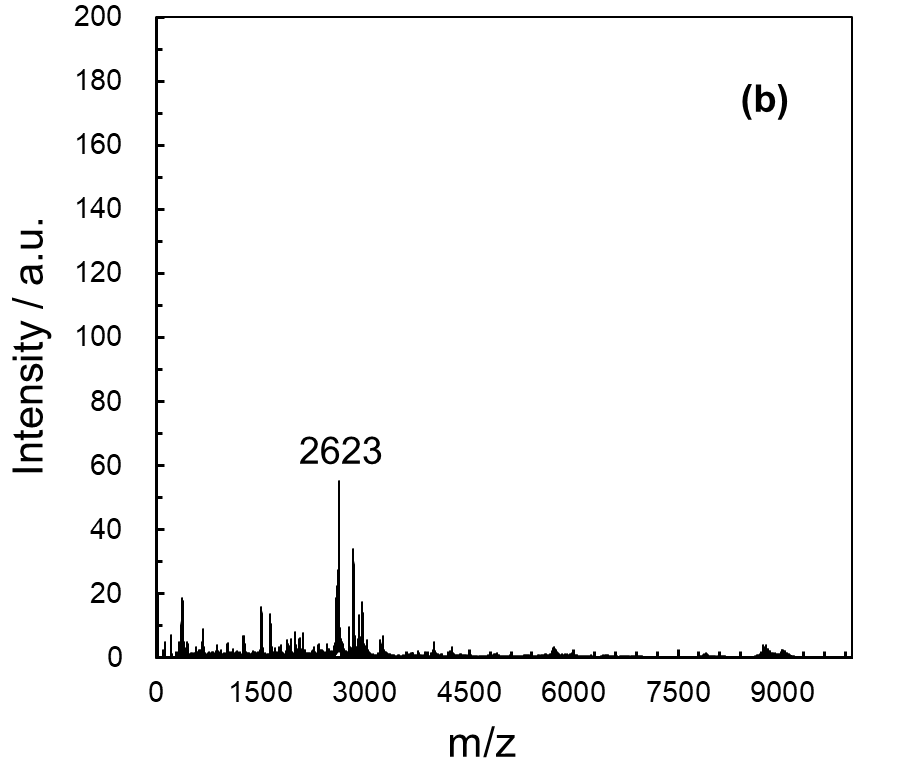


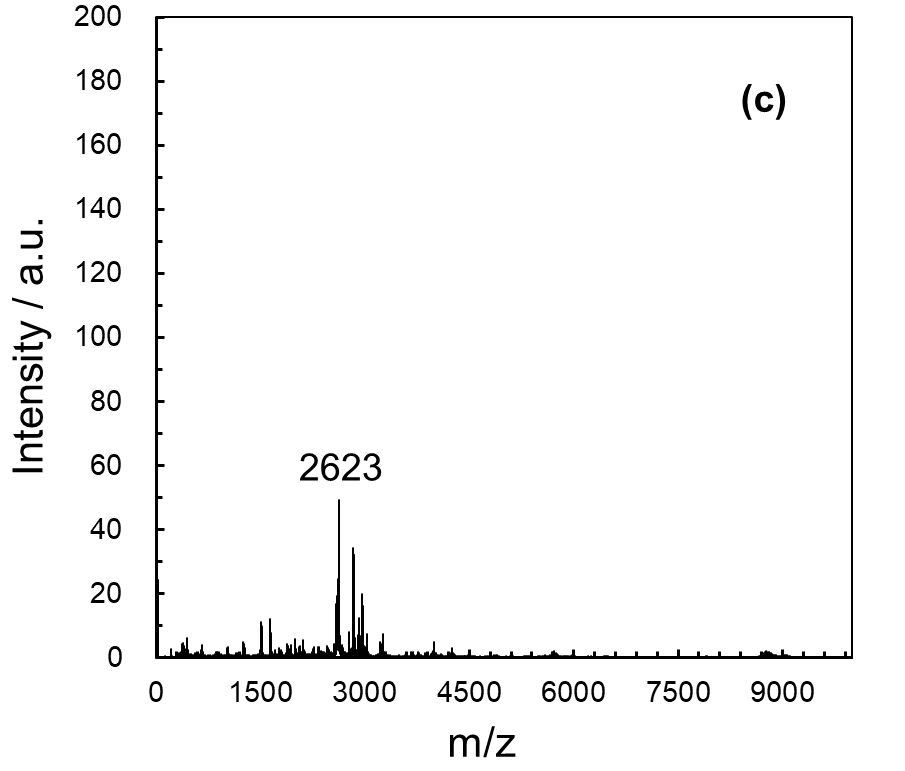

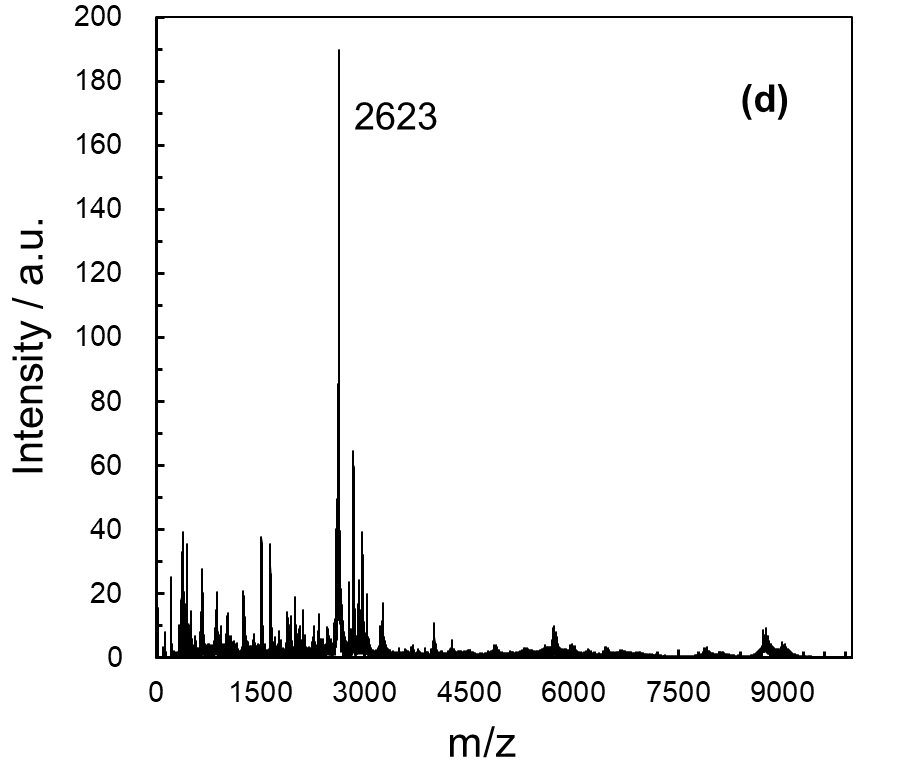


**Figure S2.** Mass spectra of the sample in the hydrolysis of casein after a 15-min heating time in the presence of the papain enzyme: (a) initial solution at 60 °C, (b) heating carried out at the maximal position of the electric field density (*E*-field), (c) heating carried out at the maximal position of the magnetic field density (*H*-field), and (d) heating carried out by the water bath method (WB).

**Table S1**. A summary of each data (sample number = 3: average, standard deviation and t-test) in Figure 2. t-test was done on water bath method (WB). Raw data in calculating the results are available to an interested reader from the corresponding author (S. Horikoshi).

|  | Average | Standard deviation |  |
| --- | --- | --- | --- |
| WB 5 min | 4.74 | 0.14 |  |
| WB 15 min | 8.60 | 0.30 |  |
| WB 30 min | 11.30 | 0.40 |  |
|  | Average | Standard deviation | t-test |
| E-field 5 min | 4.29 | 0.14 | 3.843 |
| E- field 15 min | 8.85 | 0.11 | 1.337 |
| E- field 30 min | 11.44 | 0.65 | 0.305 |
|  | Average | Standard deviation | t-test |
| H- field 5 min | 4.66 | 0.26 | 0.426 |
| H- field 15 min | 8.49 | 0.31 | 0.449 |
| H- field 30 min | 11.30 | 0.40 | 0.001 |
|  | Average | Standard deviation | t-test |
| CMI 5 min | 4.49 | 0.45 | 0.933 |
| CMI 15 min | 7.99 | 0.70 | 1.387 |
| CMI 30 min | 10.93 | 0.90 | 0.645 |
|  | Average | Standard deviation | t-test |
| PMI 5 min | 5.37 | 0.37 | 2.716 |
| PMI 15 min | 9.51 | 0.35 | 4.071 |
| PMI 30 min | 13.38 | 0.46 | 5.893 |
|  | Average | Standard deviation | t-test |
| Cooling 5 min | 4.31 | 0.28 | 2.337 |
| Cooling 15 min | 7.85 | 0.43 | 2.246 |
| Cooling 30 min | 10.52 | 1.00 | 1.249 |

**Table S2**. A summary of each data (sample number = 3: average, standard deviation and t-test) in Figure 3. t-test was done on water bath method (WB). Raw data in calculating the results are available to an interested reader from the corresponding author (S. Horikoshi).

| Arginine (Arg) | Average | Standard deviation | t-test |
| --- | --- | --- | --- |
| WB | 3677.927 | 125.831 | – |
| E-field | 3581.510 | 361.663 | 0.436 |
| H-field | 3596.597 | 107.853 | 0.850 |
| PMI | 4058.000 | 177.924 | 3.021 |
| Lysine (Lys) | Average | Standard deviation | t-test |
| WB | 2839.707 | 62.368 | – |
| E-field | 2603.037 | 128.157 | 2.876 |
| H-field | 2730.930 | 209.442 | 0.862 |
| PMI | 3226.667 | 63.359 | 7.538 |

**Table S3**. A summary of each data (sample number = 3: average, standard deviation and t-test) in Figure 4. t-test was done on water bath method (WB). Raw data in calculating the results are available to an interested reader from the corresponding author (S. Horikoshi).

|  | Average | Standard deviation |  |
| --- | --- | --- | --- |
| WB 5 min | 10.96 | 1.63 |  |
| WB 15 min | 11.15 | 0.64 |  |
| WB 30 min | 11.20 | 0.81 |  |
|  | Average | Standard deviation | t-test |
| E-field 5 min | 0.48 | 0.03 | 11.165 |
| E-field 15 min | 0.21 | 0.01 | 29.502 |
| E-field 30 min | 0.45 | 0.04 | 23.108 |
|  | Average | Standard deviation | t-test |
| H-field 5 min | 1.77 | 0.07 | 9.778 |
| H-field 15 min | 3.13 | 0.10 | 21.363 |
| H-field 30 min | 4.52 | 0.08 | 14.300 |
|  | Average | Standard deviation | t-test |
| CMI 5 min | 1.61 | 0.09 | 9.940 |
| CMI 15 min | 3.09 | 0.14 | 21.222 |
| CMI 30 min | 4.93 | 0.93 | 8.802 |
|  | Average | Standard deviation | t-test |
| PMI 5 min | 0.19 | 0.07 | 11.463 |
| PMI 15 min | 0.22 | 0.06 | 29.334 |
| PMI 30 min | 0.17 | 0.06 | 23.658 |
|  | Average | Standard deviation | t-test |
| Cooling 5 min | 1.77 | 0.06 | 9.783 |
| Cooling 15 min | 3.04 | 0.15 | 21.295 |
| Cooling 30 min | 4.52 | 0.42 | 12.753 |

**Table S4**. A summary of each data (sample number = 3: average, standard deviation and t-test) in Figure 5. t-test was done on water bath method (WB). Raw data in calculating the results are available to an interested reader from the corresponding author (S. Horikoshi).

|  | Average | Standard deviation |  |
| --- | --- | --- | --- |
| WB 0 min | 0.00 | ≈0.00 |  |
| WB 2 min | 21.53 | 0.72 |  |
| WB 3 min | 37.50 | 0.87 |  |
| WB 5 min | 49.63 | 1.01 |  |
| WB 6 min | 58.13 | 1.70 |  |
|  | Average | Standard deviation | t-test |
| CMI 0 min | 0.00 | 0.00 | – |
| CMI 2 min | 37.01 | 2.52 | 10.235 |
| CMI 3 min | 49.99 | 2.59 | 7.930 |
| CMI 5 min | 55.30 | 1.61 | 5.160 |
| CMI 6 min | 57.70 | 0.70 | 0.410 |
|  | Average | Standard deviation | t-test |
| PMI 0 min | 0.00 | 0.00 | – |
| PMI 2 min | 49.55 | 1.09 | 37.103 |
| PMI 3 min | 66.70 | 1.82 | 25.082 |
| PMI 5 min | 75.03 | 3.30 | 12.739 |
| PMI 6 min | 86.68 | 1.85 | 19.656 |
